# Baseline mapping of Oropouche virology, epidemiology, therapeutics, and vaccine research and development

**DOI:** 10.1038/s41541-022-00456-2

**Published:** 2022-03-17

**Authors:** Megan A. Files, Clairissa A. Hansen, Vanessa C. Herrera, Craig Schindewolf, Alan D. T. Barrett, David W. C. Beasley, Nigel Bourne, Gregg N. Milligan

**Affiliations:** 1 Department of Microbiology and Immunology, University of Texas Medical Branch, Galveston, TX USA; 2 Institute for Translational Sciences, University of Texas Medical Branch, Galveston, TX USA; 3 Department of Pathology, University of Texas Medical Branch, Galveston, TX USA; 4 Department of Preventive Medicine and Population Health, University of Texas Medical Branch, Galveston, TX USA; 5 Sealy Institute for Vaccine Sciences, University of Texas Medical Branch, Galveston, TX USA; 6 World Health Organization Collaborating Center for Vaccine Research, Evaluation and Training on Emerging Infectious Diseases, University of Texas Medical Branch, Galveston, TX USA; 7 Institutional Office of Regulated Nonclinical Studies, University of Texas Medical Branch, Galveston, TX USA; 8 Division of Vaccinology, Department of Pediatrics, University of Texas Medical Branch, Galveston, TX USA; 9 Present Address: Office of Public Health Outbreak Coordination, Informatics & Surveillance, Division of Disease Control and Health Statistics, Washington State Department of Health, Shoreline, WA USA

**Keywords:** Policy and public health in microbiology, Viral infection

## Abstract

Oropouche virus (OROV) is an arthropod-borne orthobunyavirus found in South America and causes Oropouche fever, a febrile infection similar to dengue. It is the second most prevalent arthropod-borne viral disease in South America after dengue. Over 500,000 cases have been diagnosed since the virus was first discovered in 1955; however, this is likely a significant underestimate given the limited availability of diagnostics. No fatalities have been reported to date, however, up to 60% of cases have a recurrent phase of disease within one month of recovery from the primary disease course. The main arthropod vector is the biting midge *Culicoides paraensis*, which has a geographic range as far north as the United States and demonstrates the potential for OROV to geographically expand. The transmission cycle is incompletely understood and vertebrate hosts include both non-human primates and birds further supporting the potential ability of the virus to spread. A number of candidate antivirals have been evaluated against OROV in vitro but none showed antiviral activity. Surprisingly, there is only one report in the literature on candidate vaccines. We suggest that OROV is an undervalued pathogen much like chikungunya, Schmallenberg, and Zika viruses were before they emerged. Overall, OROV is an important emerging disease that has been under-investigated and has the potential to cause large epidemics in the future. Further research, in particular candidate vaccines, is needed for this important pathogen.

## Discovery and classification

Oropouche virus (OROV), the causative agent of Oropouche fever (OROF), was first identified in 1955 from the blood of a febrile forest worker in Trinidad and Tobago^1^. It is a member of the *Simbu* serogroup of viruses (which also includes Akabane, Manzanilla, Sathuperi, Shamonda, Shuni, and Simbu viruses) within the *Orthobunyavirus* genus of the family *Peribunyaviridae*^2–4^. It is considered a biosafety level 2 (BSL-2) or 3 agent, depending on the specific country. For example, it is BSL-2 in the United States but BSL-3 in Australia. OROV is an arthropod-borne virus (or arbovirus), which has transmission cycles that involve both midges and mosquitoes as the arthropod hosts, and primates and birds as the vertebrate hosts^3–5^.

## Structure and genome organization

OROV is an enveloped virus with a tripartite genome composed of three single-stranded, negative-sense RNA segments^5^. The small (S) segment contains two overlapping open reading frames (ORFs), which encode the nucleocapsid protein N and the nonstructural (NS) protein NSs, a type I interferon inhibitor^6^. The medium (M) segment encodes a polyprotein that is post-translationally cleaved into the structural glycoproteins Gn and Gc, and the NSm protein^3,4^, whose function has not been determined^6^. Gc is a 939 amino acid class II membrane fusion protein with 3–4 putative N-linked glycosylation sites, and Gn is 290 amino acids with one putative N-linked glycosylation site. There is surprisingly little structural data available for Gc and Gn in members of the Orthobunyavirus genus. To date, there is only a low-resolution cryo-EM structure for Bunyamwera (BUNV) and a high-resolution crystallographic structure for the N-terminal half of Schmallenberg (SBV) Gc^7,8^. showing an elongated multi-domain protein composed of an α-helical head domain connected to a stalk region composed of two β-sheet subdomains^7^. In comparison, the structures of only the Gc a-helical head domains from BUNV, OROV, and La Crosse virus (LACV) are available, which indicate that the a-domain appears conserved by these four viruses^7^. Each has one N-linked glycosylation site that is thought to be solvent exposed. From the available data, it is believed that Gc and Gn form a trimeric spike complex protruding from the viral envelope^8^. However, no monoclonal antibodies have been generated against OROV so the location of epitopes involved in neutralization and protective immunity is unknown although the epitopes involved in protective immunity have been mapped to Gn for SBV^7^. No cell receptors have been identified for OROV.

The large (L) segment encodes the L protein and an RNA-dependent RNA polymerase^4,9^. Flanking the coding regions on each segment are untranslated regions (UTRs) important for replication, transcription, and packaging^10,11^. Although high-resolution structural studies have not been undertaken with OROV, studies with other orthobunyaviruses suggest that the enveloped OROV virion is about 90 nm in diameter, displaying Gc and Gn on the surface and containing three ribonucleoprotein (RNP) complexes—the RNA segments each complexed with many copies of N and L proteins^12^.

## Phylogeny

Orthobunyaviruses have traditionally been classified by serologic methods, such as plaque reduction neutralization test (PRNT), complement fixation, and hemagglutination inhibition (HI)^5^. However, serological approaches have gradually given way to nucleotide sequence-based approaches. Genetic studies have characterized the phylogenetic relationships of available sequences of OROV and related *Simbu* serogroup viruses^2–4,10,13,14^. OROV has been subdivided into four genotypes (I, II, III, and IV) based on analysis of the N gene, with the mean nucleotide difference among genotypes being around 5%^15^. However, more recent classification schemes, based on the sequences of all three RNA segments, suggest two clades or lineages within the M or L segment phylogenies^3,10,15^. The standardized use of four genotypes or two lineages has yet to be agreed upon. Although the first isolation of OROV occurred in Trinidad and Tobago^1^, analysis of S- and L-segment mutation rates suggests OROV may have originated in the early twentieth century in northern Brazil and has since spread northward into Trinidad and Tobago, and Panama, southward to central Brazil, and westward into Peru and Ecuador^3,15^.

## Reassortment

As the OROV genome comprises three RNA segments, reassortment among OROV genotypes or between OROV and other *Simbu* serogroup viruses is theoretically possible. The latter scenario poses a risk for the emergence of novel *Simbu* serogroup viruses. In vitro studies with a virus-like particle (VLP) system, showed OROV glycoproteins packaged the RNP of a fellow *Simbu* serogroup member SBV^16^, which is itself considered a reassortant of Shamonda and Sathuperi viruses^17^. Moreover, SBV N and L proteins were able to promote transcription of an OROV M-segment minigenome, demonstrating cross-recognition among viral components within members of the serogroup, and providing experimental evidence of the viability of reassortant viruses. Since the M segment encodes the envelope glycoproteins, which are major antigenic determinants, selective pressure could result in a higher degree of divergence and reassortment in the M segment among *Simbu* serogroup members^10^. Indeed, sites of positive selection are found within Gc, as well as motifs denoting N-linked glycosylation sites that are distinct for different OROV lineages^3^.

Importantly, there is also evidence that reassortment between *Simbu* serogroup viruses occurs naturally, with the S and L segments of one virus tending to reassort together with the M segment of another. Iquitos virus, isolated from a febrile patient in 1999 in the northeastern Amazon region of Peru, shares similarity to OROV in the S and L segments but contains a novel *Simbu* serogroup M segment, and is serologically distinct from OROV^18^. Similarly, Madre de Dios virus, isolated from a febrile patient in Peru in 2007 and from a monkey in Venezuela in 2010^4,19^, contains an M segment similar to Iquitos virus^2^, suggesting a currently unidentified *Simbu* serogroup virus exists in South America. While it remains to be seen whether OROV reassortants will pose a significant public health threat, the clinical importance of reassortants of other orthobunyaviruses has been established. For example, Ngari virus, a reassortant of Bunyamwera and Batai viruses, has been associated with hemorrhagic fever in East Africa in 1997–1998^20^. It seems highly likely that additional OROV reassortants of clinical importance will be identified in the future.

## Pathophysiology and clinical disease

Following the bite of an OROV-infected midge or mosquito^21–23^, there is a 3–8-day incubation period before disease onset^23–25^. The patient from whom the virus was first isolated (Melajo Forest, Trinidad, 1955) reported symptoms that included fever, backache, and cough without a sore throat in an illness that lasted three days with no recurring symptoms^1^. In subsequent documented cases, the symptom most commonly reported during acute disease is fever (~39 °C), which is frequently accompanied by headache/retro-orbital pain, malaise, myalgia, arthralgia, nausea, vomiting, and photo-phobia^2,26^. Less frequent symptoms include a rubella-like rash, meningitis, encephalitis, dizziness, anorexia, and other systemic manifestations^2,25,27–30^. Hemorrhagic phenomena such as epistaxis, gingival bleeding, and petechiae, or gastrointestinal manifestations such as diarrhea are even more infrequent^30^.

During the acute disease, patients experience viremia that peaks on day 2 after the onset of clinical symptoms and decreases over the next several days^31^. Elevated liver enzymes and leukopenia (values as low as 2000 leukocytes/mL) also occur^23,25^. In most people, the acute disease is relatively short, lasting from 2 to 7 days but in some, particularly those who experience central nervous system involvement (meningitis and encephalitis), the disease can last for 2–4 weeks and may include loss of strength (asthenia)^25^.

In approximately 60% of clinical cases, the disease recurs after the patient becomes afebrile (normally within 2-10 days but occasionally up to a month). Recurrence is associated with a range of symptoms including fever, headache, myalgia, asthenia, dizziness, and meningitis^32^. The mechanisms responsible for the recurring disease remain undefined. Once the patient finally recovers, there are no reported long-term sequelae or reports of additional recurrence events at a later date and, although the disease can be severe, no cases of human fatality have been reported^2,23,28,33^. Currently, it is unclear whether certain OROV genotypes are more likely to produce more severe or unusual symptoms^23,28^.

## Diagnostics and surveillance

The similarity of OROF to the infections caused by other arboviruses, such as chikungunya and dengue, which often circulate in the same areas^24^, makes the clinical diagnosis of OROV infection by symptomology or on the basis of clinical laboratory tests problematic. Therefore, the disease is sometimes misdiagnosed and consequently under-reported^33^.

Multiple diagnostic techniques are available for OROV. Because peak viremia tends to coincide with the onset of acute febrile illness^24,31^, during this time, it is possible to measure viral RNA and antigen in the blood. Additionally, patients begin to generate IgM and IgG antibodies 1 day to 2 weeks after disease onset allowing serologic testing^34^.

Historically, HI and enzyme-linked immunosorbent assays (ELISAs) have been used to measure human OROV-specific antibodies^24,25^. IgM and IgG ELISAs have been used to distinguish OROV from other arboviruses (Venezuelan equine encephalitis and dengue viruses) in febrile and afebrile Peruvian soldiers^35^. PRNTs, HI, and ELISAs have been used as surveillance tools and for epidemiological studies to determine OROV seroprevalence in livestock and wild animals^36,37^. Many serological assays require virus or virus-infected cells as a source of antigen^27,30^, necessitating appropriate biosafety facilities. Thus, the development of an immunoassay utilizing bacterially expressed recombinant N protein, (highly conserved among OROV strains), able to detect both OROV-specific IgG and IgM antibodies with a sensitivity of 95% and a specificity of 99.5% represents an important advance^38^.

A variety of reverse transcriptase–polymerase chain reaction (RT-PCR)-based diagnostic tools have been developed for OROV. The S segment has been used in several PCR-based diagnostics because it is the least variable among the *Simbu* serogroup^31,39^. However, because the S segment is so highly conserved^40^ between OROV strains and OROV-like reassortants, many RT-PCR methods cannot be considered OROV-specific^31,39,41,42^.

Some studies have used a one-step RT-PCR method in a multiplex RT-qPCR platform to detect OROV, Mayaro (MAYV), and OROV-like reassortants. The primer design for MAYV, a co-circulating alphavirus, targets the NSP1 coding region, while the OROV primers target conserved sequences of the S segment. The multiplex RT-qPCR platform is highly sensitive and can detect as few as 2–20 genome copies/mL, but the design detects both OROV and S segment-containing reassortant viruses^43^.

Rapid detection of OROV is a priority during outbreaks in isolated or rural regions. Thus, a one-step RT-qPCR method was developed to test for orthobunyaviruses using a mobile SmartCycler^TM^^44^. The primer design was based on the S segment of the prototype OROV strain TrVL 9760. The limit of detection of this diagnostic tool was determined to be 10^2^ copies/mL with a 93.3% detection rate during the first 5 days of symptoms and appears superior to the previously developed nested RT-PCR method^44,45^. In an attempt to resolve the issue of detecting both OROV and reassorted strains, RT-qPCR, RT-PCR, and nested RT-PCR methods were developed to target the M segment. These methods of quantifying OROV require further validation before they are used widely in clinical studies^13^.

Although OROV RNA has been detected in bodily fluids other than blood, there has been little systematic assessment of the efficacy of testing for the presence of OROV in bodily fluids such as cerebral spinal fluid^33^, urine, and saliva^41,46^. Detection of OROV RNA in urine and saliva may allow for the development of safe, non-invasive, simple, and effective diagnostics. Further studies are necessary to determine the limits of detection of such assays.

RT-PCR-based diagnostic platforms are best used as part of an orthogonal testing approach, in conjunction with serological testing or viral antigen detection in cases where RT-PCR methods fail or are unable to detect divergent OROV strains due to the high specificity to the target sequence^47,48^. Metagenomic analysis of patient samples has been used as a means to detect and identify divergent, reassorted, or previously unknown viral strains, where other RT-qPCR systems may fail to detect viral RNA^47^, and can further inform the optimized design of diagnostic tools^13,48^. However, metagenomic analysis is not widely available in clinical laboratories in arbovirus-endemic regions. In light of the many circulating arboviruses in South America, a comprehensive one-step multiplex RT-PCR-based diagnostic would be desirable to differentiate between OROV and other common viral illnesses endemic to the area. However, this type of assay requires specialized equipment and reagents, as well as appropriate storage of samples. Other methods, such as the ELISA to detect antibodies against recombinant OROV nucleocapsid^38^, maybe more easily adopted. Regardless of the method, there is currently no diagnostic standard for OROV, and the field would benefit from standardization to assist in the development and validation of more reliable and readily available diagnostic tools.

## Epidemiology

**Fig. 1:**
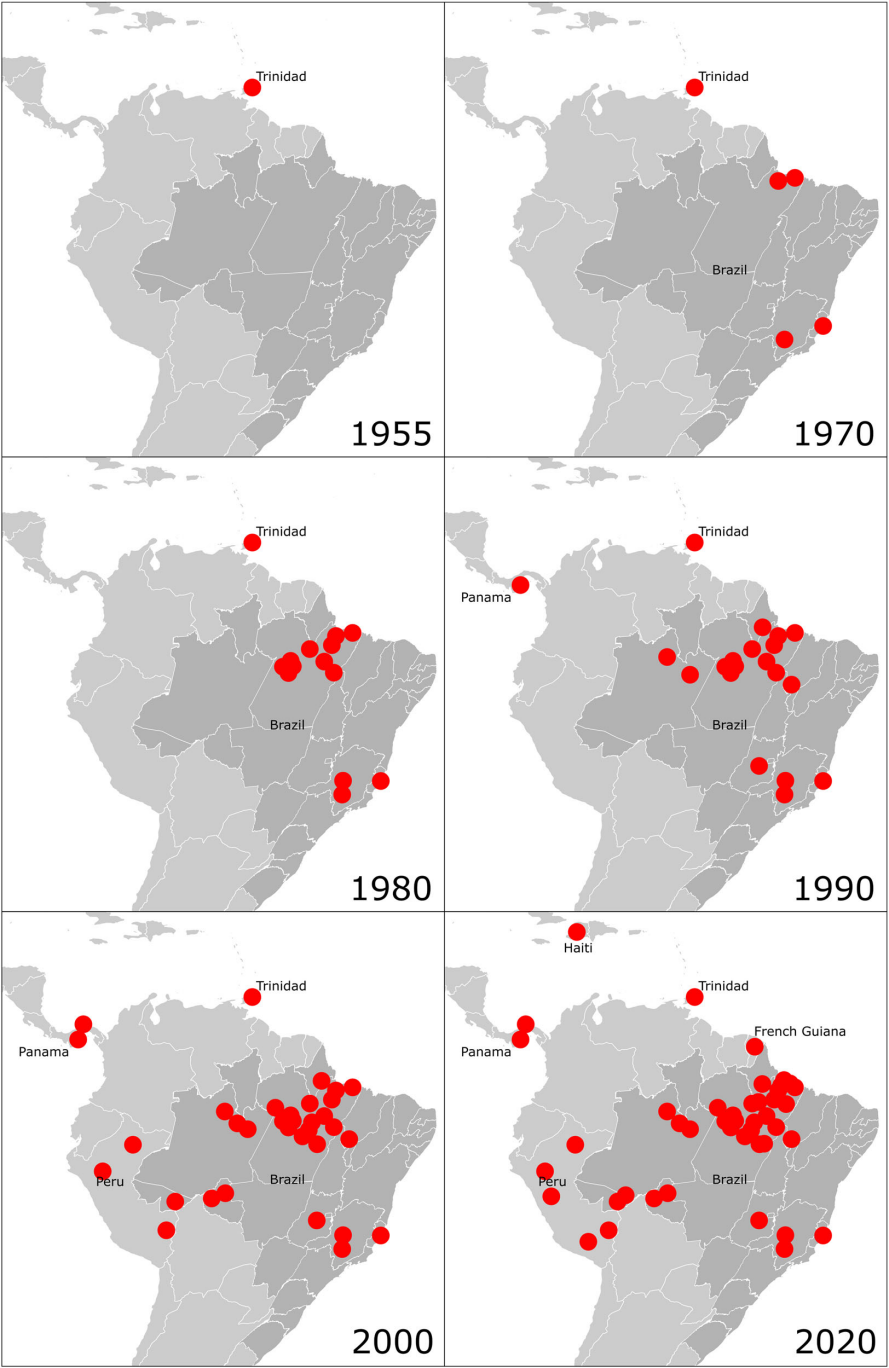
Timeline of Oropouche fever outbreaks. Red dots indicate outbreaks with serological evidence and/or confirmatory viral nucleic acid detection. Data were taken from refs. ^1–3,52,54,94^. Map graphics adapted from ref. ^95^.

Following its isolation in Trinidad in 1955^1^, OROV was next seen clinically in 1961, in an epidemic in Belém, Brazil that involved an estimated 11,000 people. Peru experienced its first epidemic in the city of Iquitos in 1992^2^. The virus has also been found in Central America where it was first isolated in 1989 from febrile patients in Panama during a dengue surveillance program^2^. Subsequently, epidemiologic surveillance has shown OROV circulation in other South American countries, including Bolivia, Ecuador, Colombia, and Venezuela^19,49–51^, and more recently in Haiti in 2014^52^ and French Guiana in 2020^53^. A description of OROF outbreaks by location has been published elsewhere^54^. and is shown pictorially over time in Fig. 1. The majority of reported cases continue to occur in the Amazon region of Brazil and Peru. Since 1961 there have been over 30 epidemics of OROF in Brazil, all in the Amazon region^55,56^. The largest epidemic to date occurred in Manaus, Brazil in 1980 resulting in an estimated 97,000 cases (approximately 15% of the population)^57^. Seven urban epidemics between 1961 and 1978 in Pará, Brazil were analyzed and found to have a common attack rate of approximately 30%^56^. More than half a million people residing in the Amazon region of Brazil alone are estimated to have been infected with OROV^56^. It is suspected that the incidence and disease burden of OROV is underestimated due to the similarity of its clinical presentation to febrile illnesses caused by other arboviruses, such as dengue, Zika, chikungunya, and yellow fever. Current surveillance for OROV is limited and the majority of surveillance methods focus on serological testing in humans and animals^25,56,58^.

**Fig. 2:**
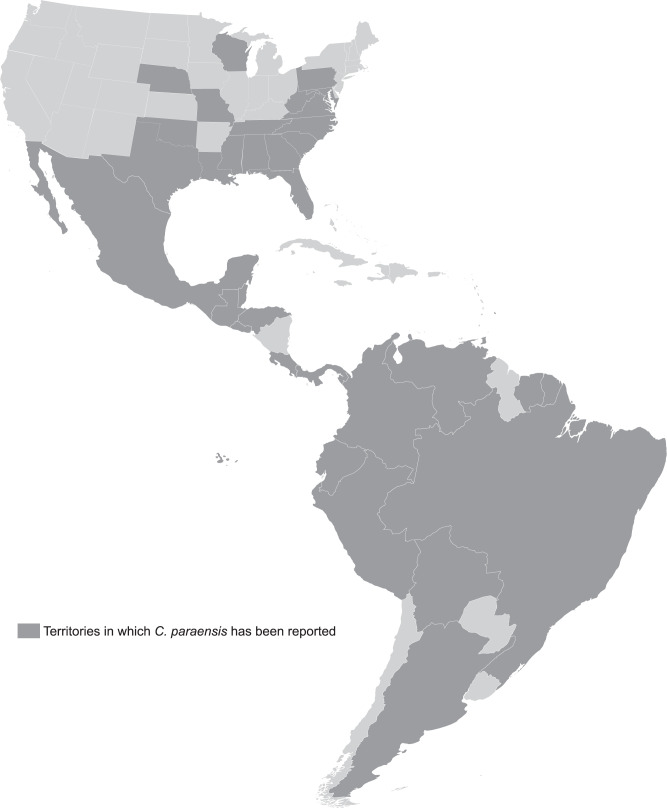
Distribution of *Culicoides paraensis* in the Americas. Dark gray indicates countries and states where *C. paraensis* has been reported^61,96–101^. Map template modified from ref. ^102^.

OROV is maintained in nature through two transmission cycles: an urban cycle and a sylvatic cycle. The urban cycle, which is generally associated with explosive outbreaks of disease, is believed to primarily involve the biting midge *Culicoides paraensis*^25,59^. Moreover, epidemics of OROF in Brazil have a seasonal pattern and have predominantly occurred during the rainy season (January to June) which is associated temporally with the highest density of *C. paraensis* populations^56,60^. Humans are believed to be the only vertebrate host in the urban cycle since OROV infection has not been detected in domestic animals, except birds. Known arthropod vectors for OROV besides *C. paraensis* are the mosquito species *Culex quinquefasciatus*, *Coquillettidia venezuelensis*, *Mansonia venezuelensis*, and *Aedes serratus*^1,25,56^. *C. paraensis* is the main vector in Brazil but has a wide geographic range that extends from Argentina and Chile to include large areas in the United States (see Fig. 2)^61^. A recent study evaluated vector competence of the prototype OROV strain TrVL 9760 in three North American vector species: *Cx. tarsalis, Cx. quinquefasciatus*, and the midge *C. sonorensis*^62^. Both mosquito species were relatively poor vectors whereas *C. sonorensis* showed high infection and dissemination, and good transmission potential. Thus, there is potential for OROV to emerge across a wide geographic area. Precedence for emergence can be seen with the related orthobunyavirus, SBV, an important veterinary pathogen in ruminants such as sheep and cattle that is also spread by *Culicoides* midges^63^. SBV emerged in Germany, the Netherlands, and Belgium in 2011 and has since spread through much of Europe^64^.

The main vertebrate hosts involved in the sylvatic cycle have not been fully identified, but there is evidence that wild birds, the three-toed sloth (*Bradypus tridactylus)*, and certain species of New World non-human primates (NHP)—principally capuchin and howler monkeys—are involved^10,25,50^. Among wild birds, HI antibodies were detected in members of the families *Formicariidae*, *Troglodytidae*, *Cuculidae*, *Fringillidae*, *Dendrocolaptidae*, *Tyrannidae*, *Vireonidae*, *Thraupidae*, and *Pipridae*^65^. Additionally, HI antibodies to OROV have been found in domestic chickens and one duck^65^. A variety of domestic vertebrates were tested for the presence of antibodies to OROV during five epidemics in Brazil. No OROV-specific antibodies were detected in cats, dogs, or pigs^25,56^.

All four of the OROV genotypes have been detected in Brazil. OROV isolates recovered from outbreaks in Brazil revealed that genotype I is more prevalent in the eastern Amazon region and genotype II is more prevalent in the western Amazon region^15,40,51^. Genotype III was originally thought to be restricted to Panama, but was detected in Minas Gerais State, southeastern Brazil in 2000^15,50^. Genotype IV appears to be restricted to Amazonas State, Brazil^15^.

Environmental and climate changes, as well as deforestation for agriculture and urbanization are predicted to be drivers for the emergence of arboviral epidemics and re-emergence of yellow fever, Mayaro, and OROV^10,60,66,67^. In Cusco, Peru, areas with vegetation loss were common locations for recent outbreaks of OROF^68^. However, ecological niche models suggested this observation could also be due to favorable transmission conditions coinciding in neighboring areas^68^. One study from Brazil examined the effects of construction and flooding on the transmission of sylvatic arboviruses. The construction of a dam in the 1980s flooded large areas in the Tucuruí area of Pará State for a three-month period. Over one million mosquitoes were captured, and a significant number of new viruses were isolated. While only one strain of OROV was isolated, there were high annual rates of OROV antibody-positive birds and NHPs during the flooding period, creating suitable ecological conditions for transmission. Prevalence rates were between 2 and 7.1% for birds, and 8.5% for monkeys^69^. More research is urgently needed to assess the relationship between landscape changes, the emergence of OROV, and other arboviral epidemics.

OROV has spread from the Amazon and is making its way into urban areas of endemic South American countries. Current prevention strategies focus on the control and reduction of arthropod vectors mainly by the elimination of vector breeding sites with chemical insecticides. Education efforts emphasizing the importance of topical insecticides and having personal protective products such as bug nets are also being employed^56^. However, the effectiveness of this individual approach may be limited by a lack of financial resources and access to these materials. Further investigation is needed to fully comprehend the epidemiology of OROV. Additionally, better surveillance methods and determination of incidence are needed to understand the exact burden OROV has on Central and South American countries.

## Animal models

OROV infection has been examined in a number of small animal models. Suckling and adult white Swiss mice inoculated intracranially (IC) with a high dose of the virus were highly susceptible to disease and all animals succumbed within 3 days. Lower doses also produced uniform lethality but with an extended incubation period. Intraperitoneal (IP) inoculation with the high dose of virus was lethal to infant mice but induced no clinical signs in adult mice^1^.

Although immunocompetent adult mice have proven resistant to disease following peripheral OROV challenge, more recent studies using adult animals of a number of immune gene knockout (KO) strains have produced lethality. The most thoroughly studied are strains involving KOs in innate immune sensing mediators associated with the RIG-I-like receptors. Specifically, the importance of innate immune sensing is suggested by hepatic injury and reduced survival of mitochondrial antiviral signaling protein knockout (MAVS^−/−^) mice compared to wild types (~40% compared to 0% mortality, respectively)^70^. On examination of downstream signaling events, interferon (IFN) regulatory factor 3 and IFN regulatory factor 7 (IRF3^−/−^ IRF7^−^^/^^−^) double knockout (DKO) mice experienced lethal infection; whereas, individually, the IRF3 and IRF7 KO models showed >90% survival. Interestingly, OROV-infected IFN regulatory factor 5 KO (IRF5^−/−^) mice exhibited signs of hepatic injury early in the infection, increased viral titers in the spinal cord, and delayed death relative to IRF3^−/−^ IRF7^−/−^ DKO mice^71^. OROV-infected IFN-β^−/−^ mice lost weight and exhibited a lethality rate of 17%, suggesting that IFN-β plays some role in protection against OROV infection in mice. However, the lower rates of lethality in IFN-β^−/−^ mice and IRF3^−/−^ IRF7^−/−^ DKO mice (~50%) in comparison to IFN-α/β receptor KO (IFNAR^−/−^) mice (100% lethality) suggest that IFN-β alone is not solely responsible for the protection and that IFN-α may be critical in controlling OROV infection^70^. In mice that lacked intact type I IFN signaling (IFNAR KO, MAVS KO, IRF3 KO, IRF7 KO), OROV titer was highest in the liver, spleen, and blood and caused extensive liver damage (dead cells, hemorrhages, tissue discoloration)^70,71^.

Hamsters have also been used to model OROV pathogenesis. Anderson et al.^1^ showed that OROV caused disease in adult Syrian golden hamsters (*Mesocrisetus auratus*) after either IC or IP inoculation. Signs of disease using either route of inoculation included loss of appetite, coat ruffling, and difficulty walking, with some animals showing complete hind limb paralysis. Virus titers in the brain at death (10^5^ units/0.02 mL) were similar using either route of inoculation^1^. Subsequently, a subcutaneous (SC) challenge model was developed in 3-week-old hamsters that resulted in over 50% of the animals developing the severe disease (lethargy, ruffled fur, weight loss, shivering, walking difficulty, paralysis) and approximately one-third succumbed. High titers of OROV were present in the blood, liver, and brain (10^2^–10^7^ TCID_50_/mL or /g). Notably, viral antigen was strongly associated with neurons. This was accompanied by histopathological signs of meningoencephalitis (microglial nodules, infiltrating mononuclear cells, perivascular cuffs with leukocytes) and hepatitis (infiltrating mononuclear cells, eosinophils, necrosis, Kupffer cell activation). In the liver, the viral titer remained higher than 10^4^ TCID_50_/g of tissue from day 3 until day 11 post-infection, suggesting highly efficient replication of the virus in this organ^72^. Overall, following infection by the SC route, hamsters show systemic infection, neurological motor impairment, paralysis, and virus accumulation in the brain and liver. They also develop fatal hepatitis, necrosis of hepatocytes, and Kupffer cell hyperplasia. It has been hypothesized that macrophages capture OROV and transfer it to hepatocytes^72^. Hepatic lesions were seen 6 h post-inoculation^73^. The fact that the virus caused these liver manifestations even after only being introduced via the IC route signifies that this virus may have very specific tissue tropism for the liver^1^.

To date, there are no reports of well-characterized NHP models of OROV infection. Serological surveys show that multiple New World NHPs, including capuchin and howler monkeys, have OROV antibodies suggesting they are susceptible to infection^1^. However, there are no reports of overt clinical signs of illness in New World NHPs infected with OROV experimentally and there have been no investigations of the use of Old World NHPs (baboons and macaques).

## Immunology

OROV infection results in the production of type I IFN in multiple mouse strains^70,71,74^. The high mortality in IFNAR KO mice demonstrates that protection against lethal infection in mice is dependent on appropriate IFNAR signaling. When IFNAR signaling was selectively knocked out in granulocytes and dendritic cells, infection was not lethal, suggesting that subsets of non-myeloid cells are predominantly responsible for producing protective IFN. In fact, in mice where IFNAR signaling was ablated in granulocytes, there was an improved survival rate, suggesting that perhaps IFN signaling in granulocytes may play a role in OROV pathogenesis^70^.

In one of the few immunological studies in humans, IFN-α was shown to be a universal biomarker of OROV infection evidenced by high levels of expression regardless of OROV antibody titer^34^. This study also showed that the cytokine profiles exhibited by early and late seroconverters were highly divergent. Early seroconverters were defined as patients with high titers of IgM and IgG 1–7 days after disease onset while late seroconverters developed high titers ≥8 days after disease onset. Early seroconverters expressed high levels of interleukin (IL)-5, presumably resulting in the differentiation of antibody-producing plasma cells earlier in infection. Early seroconverters also expressed high levels of C-X-C motif chemokine ligand (CXCL) 8, known to recruit neutrophils and other granulocytes; however, the role of neutrophilic activity in OROV pathogenesis has yet to be characterized. Late seroconverters expressed high levels of IL-17 and CXCL10 (also known as IP-10). All patients demonstrated increased levels of IFN-α and CXCL8, and decreased levels of tumor necrosis factor (TNF) and IL-10. Regardless of the association between seroconversion and cytokine profile, there was no correlation between time of seroconversion and viremia or symptomology^34^.

While type I IFN responses appear essential to combat OROV infection, the IFN-stimulated genes responsible for the restriction of OROV replication remain to be fully elucidated. There are few studies that explore the host response to OROV infection, and even fewer that utilize patient samples. There are currently no studies following patients who have cleared OROV infection, thus the adaptive immune response to OROV infection is poorly understood. Furthermore, questions regarding the role of T cells in the pathogenesis or control of OROV and the kinetics of B cell activation, differentiation, and OROV-specific antibody production remain unanswered.

## Antivirals

Currently, there is no specific antiviral therapy for OROV infection, and existing literature describing possible treatments for OROF is limited. Ribavirin (RBV), mycophenolic acid (MPA), and IFN-α have been investigated for OROV treatment. In in vitro studies, RBV showed no antiviral activity against OROV infection but was active against two other orthobunyaviruses (Tacaiuma virus (TCMV) and Guama virus (GMAV))^75^. RBV had no antiviral activity against OROV or any of the other orthobunyaviruses tested in newborn Swiss mice^75^. Similarly, in vitro administration of MPA had no antiviral activity against OROV but was active against GMAV and TCMV^76^. IFN-α showed limited activity in vitro that was dependent on the dose and timing of treatment^77^. In vivo, mice dosed IP route with 30 μL of IFN-α-2a per day for 10 days beginning 1 day before infection survived a lethal OROV challenge. When treatment was initiated 3 or 24 h after infection the protective effect on mortality was lost and viral replication in the brain was not prevented^77^.

The drug favipiravir, a nucleoside analog, has not been tested against OROV, but shows promising activity against multiple viruses belonging to the family *Peribunyaviridae* suggesting such studies are warrented^78,79^.

## Vaccines

To date, there has been only one published preclinical or clinical study on candidate OROF vaccines, other than an immunoinformatic analysis that identified a number of putative T and B cell epitopes within the OROV M-segment polyprotein^80^. Recently, a candidate vaccine based on replication-competent vesicular stomatitis virus (VSV) expressing the OROV glycoproteins was shown to protect mice from wild-type challenge. Briefly, C57BL/6 mice were given two doses of 10^6^ focus forming units (ffu) of rVSV-OROV by the SC route, 28 days apart, and challenged 7 days later with 10^6^ TCID_50_ OROV strain BeAn19991 by the SC route. Protection was shown by no loss of body weight or increase in body temperature, and reduced viral loads in vaccinated mice compared to control mice^81^.

The recent development of a reverse genetics system for OROV should prove beneficial to vaccine development^6^. Vaccine development efforts for OROF are likely to be guided by approaches taken with other orthobunyaviruses of clinical and veterinary interest where live attenuated, chemically inactivated, DNA-vectored, and protein-subunit immunization strategies have been employed. Attenuation strategies include deletion of the nonstructural proteins^82,83^ or parts of the UTRs^11^, swapping of UTRs between segments^84^, swapping of the M segment coding regions of related orthobunyaviruses^85^, and mutation of N^86^.

OROF vaccine development may also be informed by attempts to develop vaccines against other members of the *Simbu* serogroup^87–89^. A number of these viruses, particularly SBV, Aino, and Akabane (AKAV) viruses are important veterinary pathogens. A binary ethylenimine (BEI)-inactivated SBV vaccine containing a pre-inactivation titer of about 10^6^ TCID_50_/mL is approved in the European Union for IM administration to cattle and SC administration to sheep. Two immunizations with this vaccine reduced (in cattle) or prevented (in sheep) viremia when animals were challenged with infectious serum 2 weeks after the second immunization^90^.

A live attenuated SBV candidate vaccine lacking both NSs and NSm, when administered SC in a single 10^6^ TCID_50_/mL dose (volume unspecified), was protective in cattle, preventing clinical signs and detectable viral load in the serum of previously SBV-naive calves after either immunization or challenge with infectious cattle serum, and by neutralizing antibody titers of at least 5 by 7 days post-challenge^83^. A bivalent SBV-AKAV protein-subunit candidate vaccine consisting of the N-termini of the Gc proteins from both SBV and AKAV, expressed in HEK-293T cells and covalently-linked has also been developed. When this vaccine was administered SC in two 50 μg doses three weeks apart, it was found to protect against challenge with a cattle-passaged field strain of SBV. Animals showed no clinical signs or detectable viral RNA in the blood or organs and neutralizing antibody titers were at least 20 at the time of challenge^91^. Two candidate DNA-vectored vaccines, encoding either N or the ectodomain of Gc, were protective in IFNAR^−/−^ mice preventing weight loss and reducing viremia compared to mock-vaccinated controls^92^. While neither vaccine candidate induced detectable neutralizing antibody, the N vaccine induced high levels of SBV-specific binding antibody and the Gc vaccine-induced proliferation of SBV-specific CD8+ T cells.

## Conclusions

OROV is a significant public health problem producing ongoing endemic disease and periodic outbreaks in substantial areas of South America. Further, there is clear potential for the virus to emerge or undergo reassortment in new geographic areas, increasing the population at risk for infection. Despite this, it is clear that comparatively little is known about the virus and there are at present no candidate medical countermeasures, other than one study of a candidate vaccine in a mouse model. This may in part be because virus infection is not lethal. There are comparatively large, active vaccine and drug research programs for Crimean-Congo hemorrhagic fever, Powassan, Nipah, and Middle Eastern respiratory syndrome (MERS) viruses—all of which cause fewer clinical cases than OROV but which are responsible for fatal infections. Nonetheless, it should be remembered that three other arboviruses have emerged to cause high morbidity with little mortality. Mosquito-borne Zika virus is associated with permanently disabling illness and some mortality in neonates, while mosquito-borne chikungunya virus is another example of a debilitating but non-lethal arboviral disease that has emerged globally in the recent past^92^. Both viruses rapidly emerged into global public health problems. Similarly, *Culicoides* midge-borne SBV rapidly emerged in Europe to become a major veterinary problem^64^. Of particular note is that the transmission cycle of OROV is incompletely understood and that the geographic range of the arthropod vectors *C. paraensis* and *C*. *sonorensis* includes North America. Additionally, the potential of birds as vertebrate hosts, raise concerns for the potential emergence of OROV in different geographic locations. Further, the proven ability of OROV and closely related viruses to produce reassortants represents a poorly defined public health threat.

The lack of publications on the evaluation of candidate antivirals against OROV is a concern as is only one report on candidate vaccines. Other bunyaviruses have had their envelope glycoproteins expressed using different vaccine platform technologies; thus, such platforms should be able to be translated to candidate OROV vaccines with relatively little effort. There is insufficient information to justify a preventative vaccine program, although this may change with improved diagnostics. Thus, a reactive vaccine program is considered more applicable.

Perhaps the most significant deficiency at this time is the lack of a characterized NHP model to evaluate candidate antivirals and vaccines for efficacy. The fact that serologic surveys indicate that multiple New World primate species are susceptible to infection provides evidence that the development of such models is possible. To date, the clinical impact of OROV has not driven the development of such models. However, this would appear warranted with the potential for OROV to become a significant public health pathogen.

Overall, OROV has the potential to become a major public health problem in an expanded geographic area, and there is an urgent need to evaluate medical countermeasures in case the virus emerges as Zika virus did a few years ago^93^.
